# Predictive value of Ki-67 expression in predicting pathological response to neoadjuvant chemotherapy combined with immunotherapy in lung squamous cell carcinoma

**DOI:** 10.3389/fonc.2026.1859319

**Published:** 2026-07-07

**Authors:** Yongliang Niu, Junguo Li, Bowen Ding, Haitang Yang, Hui Zhao, Diming Wang

**Affiliations:** 1 Department of Respiratory and Critical Care Medicine, Second Affiliated Hospital of Anhui Medical University, Hefei, Anhui, China; 2 Department of Respiratory and Critical Care Medicine, No.2 People’s Hospital of Fuyang City, Fuyang Infectious Disease Clinical College of Anhui Medical University, Fuyang, China; 3 Department of Pathology, Linquan County People’s Hospital, Fuyang, Anhui, China; 4 Department of Pathology, Shanghai Chest Hospital, Shanghai Jiao Tong University School of Medicine, Shanghai, China; 5 Department of Thoracic Surgery, Shanghai Chest Hospital, Shanghai Jiao Tong University School of Medicine, Shanghai, China; 6 Department of Oncology, Anhui Chest Hospital, Hefei, China

**Keywords:** Ki-67 expression, lung squamous cell carcinoma, major pathological response, neoadjuvant chemoimmunotherapy, predictive biomarker

## Abstract

**Background:**

Neoadjuvant chemoimmunotherapy is standard for locally advanced lung squamous cell carcinoma (SCC), but reliable predictive biomarkers for pathological response are lacking. Ki-67 has shown promise in advanced disease, yet its role in the neoadjuvant setting remains undefined.

**Methods:**

We retrospectively analyzed 137 patients with stage IIIA–IIIB lung SCC who received neoadjuvant PD-1 inhibitors plus platinum−based chemotherapy followed by surgery (2022–2025). Major pathological response (MPR) and pathological complete response (pCR) were assessed per IASLC criteria and combined as MPR/pCR. Ki-67 expression was measured by immunohistochemistry (clone UMAB107). ROC curve, univariate and multivariate logistic regression were used to evaluate predictive performance.

**Results:**

MPR/pCR was achieved in 77/137 patients (56.2%). The optimal Ki-67 cutoff was 55% (AUC = 0.785, 95%CI: 0.707–0.862, P<0.001). High Ki-67 (≥55%) was associated with a significantly higher MPR/pCR rate than low Ki-67 (69.6% vs. 27.9%, P<0.001). In multivariate analysis, Ki-67 expression (adjusted OR per 1% increase =1.069, 95%CI: 1.043–1.096, P<0.001) and smoking status (adjusted OR = 5.405, 95%CI: 1.515–19.280, P = 0.009) were independent predictors of MPR/pCR. PD-L1 expression showed no significant association (P = 0.607).

**Conclusions:**

Pretreatment Ki-67 expression (optimal cutoff 55% in this cohort) is a robust independent predictor of pathological response to neoadjuvant chemoimmunotherapy in locally advanced lung SCC. Smoking status is also an independent predictor. Ki-67 assessment may help identify patients likely to benefit from neoadjuvant therapy, but prospective multi−center validation is required before routine clinical application.

## Introduction

In recent years, perioperative immunotherapy has significantly improved outcomes for patients with resectable non-small cell lung cancer (NSCLC). The CheckMate 816 trial first demonstrated that neoadjuvant nivolumab plus chemotherapy significantly improved pathological complete response (pCR) rates (24.0% vs. 2.2%) and event-free survival (EFS) compared with chemotherapy alone (1). Building upon this, the RATIONALE-315 and Neotorch trials further expanded the application of perioperative immunotherapy, showing that tislelizumab or toripalimab plus neoadjuvant chemotherapy achieved major pathological response (MPR) rates of 56% and 48.5%, respectively, markedly superior to chemotherapy alone (2, 3). Neoadjuvant chemoimmunotherapy demonstrates significantly superior pathological response rates compared with chemotherapy alone (historical pCR rates: 5–8%) and single-agent immunotherapy (MPR rates: 14–45%), achieving MPR rates of 46–83% (4).These landmark trials have established immune-chemotherapy combinations as the standard neoadjuvant regimen for resectable NSCLC.

Pathological response (including MPR and pCR) has emerged as a critical surrogate endpoint for evaluating treatment efficacy and predicting long-term survival in the neoadjuvant setting. However, reliable biomarkers capable of predicting pathological response before treatment initiation remain lacking. While Ki-67 remains a well-established marker of tumor proliferative activity, contemporary oncology is increasingly moving towards integrated molecular approaches based on genomics, transcriptomics, proteomics, pharmacogenomics and other multi-omics technologies. Ki-67 may ultimately complement rather than replace these more comprehensive biomarker strategies. Nevertheless, identifying accessible and reproducible protein-level biomarkers such as Ki-67 remains clinically valuable, especially in resource-limited settings where high-throughput omics are not routinely available. The identification of such predictive markers holds significant clinical value for selecting optimal candidates and avoiding ineffective treatment.

Ki-67, a nuclear protein associated with cellular proliferation, serves as a classic marker of tumor proliferative activity. Studies by Wang et al. (5, 6) in advanced squamous cell lung carcinoma demonstrated that high Ki-67 expression correlates with improved objective response rates to platinum-based chemotherapy yet paradoxically predicts shorter progression-free and overall survival. Extending into the immunotherapy era, recent evidence suggests Ki-67 may further stratify treatment efficacy in immune-chemotherapy combinations. Yang et al. (7)demonstrated in PD-L1-high advanced NSCLC that Ki-67 >30% identified patients deriving significant survival benefit from chemo-immunotherapy versus immunotherapy monotherapy (HR 0.47), whereas Tao et al. (8) identified Ki-67 as an independent predictor of immunotherapy response within a machine learning model incorporating tumor stage and TMB. Notably, the predictive value of Ki-67 for pathological response (particularly MPR/pCR) in resectable squamous NSCLC undergoing neoadjuvant immunotherapy remains unclear.

Therefore, the present study aims to evaluate the predictive value of Ki-67 expression for pathological response (MPR/pCR) in patients with resectable squamous cell lung carcinoma receiving neoadjuvant immunotherapy plus chemotherapy, and to explore its potential clinical application as a biomarker for perioperative immunotherapy efficacy.

## Methods

### Study population and design

We conducted a retrospective analysis of consecutive patients with histologically confirmed stage IIIA–IIIB (according to the 8th edition of the TNM Classification for Lung Cancer) lung squamous cell carcinoma (SCC), who underwent neoadjuvant therapy followed by surgical resection at Shanghai Chest Hospital between 2022 and 2025. The inclusion criteria were as follows: (1) pathologically confirmed lung SCC; (2) clinical stage IIIA–IIIB; (3) receipt of three cycles of neoadjuvant paclitaxel (175 mg/m²), or nab-paclitaxel (260 mg/m², d1) and carboplatin (AUC 5–6) combined with PD-1 inhibitors; (4) subsequent surgical resection (lobectomy or pneumonectomy with systematic lymph node dissection); (5) availability of complete clinical data and pretreatment tumor tissue samples for immunohistochemistry analysis; (6) an Eastern Cooperative Oncology Group (ECOG) performance status score of 0–1. Exclusion criteria included: (1) prior antitumor therapy (chemotherapy, radiotherapy, or targeted therapy); (2) mixed histology or non-squamous NSCLC; (3) incomplete resection (R1/R2); (4) absence of pretreatment biopsy samples;(5) active autoimmune diseases or severe comorbidities that contraindicate immunotherapy. This study was conducted in accordance with the Declaration of Helsinki and approved by the Ethics Committee of Shanghai Chest Hospital (approval number:IS24185). Given the retrospective nature, the requirement for informed consent was waived.

Patients with clinical stage IIIB disease were carefully selected for surgery based on the following criteria: (1) single-station N2 involvement without bulky mediastinal disease (defined as lymph node short-axis diameter < 2 cm on pretreatment imaging); (2) significant downstaging (to ≤N1 or N2 with marked nodal shrinkage) after three cycles of neoadjuvant chemoimmunotherapy, as assessed by restaging CT and/or PET-CT; (3) multidisciplinary team consensus that complete (R0) resection was technically feasible. No patient with multistation N2 or T4 disease with mediastinal invasion underwent upfront surgery without evidence of downstaging.

### Neoadjuvant treatment and surgical intervention

All patients received three cycles of neoadjuvant immunotherapy combined with chemotherapy. The immunotherapy regimens included pembrolizumab (200 mg, Q3W), toripalimab (240 mg, Q3W), or other PD-1 inhibitors, administered intravenously on day 1 of each cycle. Chemotherapy consisted of paclitaxel (175 mg/m²) or nab-paclitaxel (260 mg/m², d1) plus carboplatin (AUC 5–6), administered every 3 weeks for three cycles. Surgery was performed 3–6 weeks after the completion of neoadjuvant therapy. Surgical procedures included lobectomy, bilobectomy, or pneumonectomy with systematic mediastinal lymph node dissection. The extent of resection was determined based on tumor location, size, and clinical staging.

### Pathological assessment

Postoperative pathological specimens were independently reviewed by two experienced thoracic pathologists who were blinded to all clinical data and treatment outcomes. Pathological response was evaluated in accordance with the criteria established by the International Association for the Study of Lung Cancer (IASLC). Major pathological response (MPR) was defined as ≤10% residual viable tumor cells in the primary tumor, regardless of lymph node status. Pathological complete response (pCR) was defined as no residual viable tumor cells in both the primary tumor and all sampled regional lymph nodes. Patients who failed to achieve MPR were defined as non-MPR. For the purpose of this study, MPR and pCR were combined into a single MPR/pCR group and compared with the non-MPR group.

### Immunohistochemistry and Ki-67 analysis

Pretreatment formalin-fixed paraffin-embedded (FFPE) tumor tissues obtained from core needle biopsy or bronchoscopic biopsy were used for immunohistochemical staining. In patients with mediastinal lymph node involvement, endobronchial ultrasound-guided transbronchial needle aspiration (EBUS-TBNA) was routinely performed for both diagnostic and staging purposes. However, due to the small amount of tissue obtained by EBUS-TBNA, which is often insufficient for reliable Ki-67 immunohistochemistry, the Ki-67 analysis in this study was primarily performed on core needle biopsy or bronchoscopic biopsy samples from the primary tumor.Ki-67 staining was performed using an automated Dako Omnis staining system with antibody clone UMAB107, following the manufacturer’s standard protocols. PD−L1 expression was analyzed using the 22C3 pharmDx assay (Dako), and tumor proportion score (TPS) was calculated as the percentage of viable tumor cells with partial or complete membranous staining.

**Figure 1:**
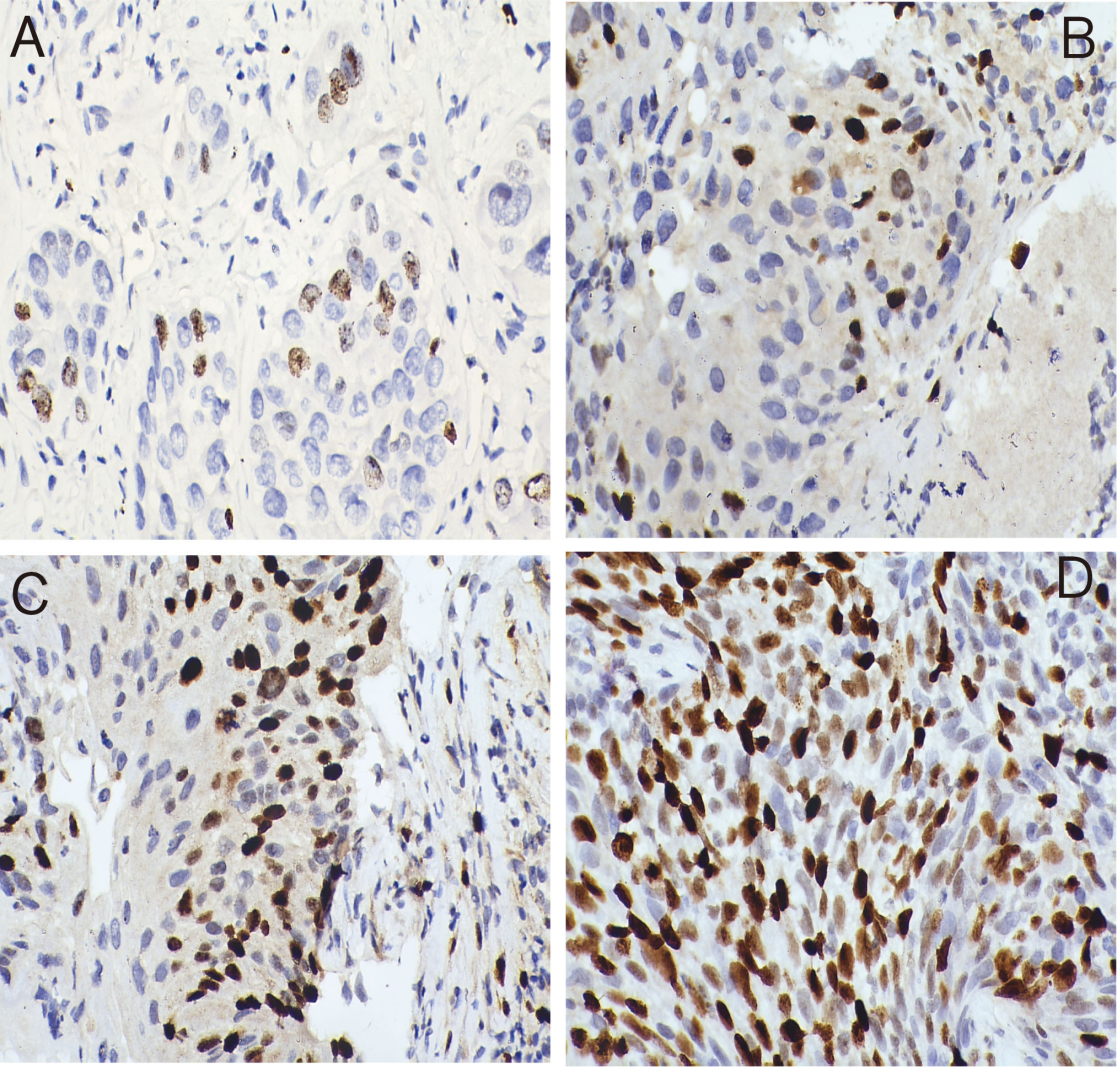
Representative immunohistochemical staining of Ki−67 in lung squamous cell carcinoma (original magnification ×400). **(A)** Low Ki−67 proliferation index; **(B)** Moderately low Ki−67 proliferation index; **(C)** High Ki−67 proliferation index; **(D)** Very high Ki−67 proliferation index.

Ki−67 proliferation index was determined as the percentage of positively stained tumor cell nuclei. All evaluations were independently performed by two experienced thoracic pathologists who were blinded to clinicopathological data and treatment outcomes. At least five randomly selected high-power fields (×400 magnification) were examined, and only viable tumor cells were counted; necrotic tissues, inflammatory cells, and stromal cells were excluded. Discrepancies between the two pathologists were resolved by consensus discussion. The Ki−67 index was recorded as a continuous percentage value for subsequent statistical analysis (**Figure 1**).

### Statistical analysis

Continuous variables were expressed as mean ± standard error of the mean (SEM), and categorical variables were presented as frequencies (percentages). The optimal cutoff value for Ki-67 expression was determined using ROC curve analysis based on the Youden index (maximum sensitivity+specificity−1). The area under the curve (AUC) with 95% confidence interval (CI) was calculated to assess the predictive accuracy of Ki-67 for MPR/pCR.

The association between Ki-67 expression and clinicopathological characteristics was assessed using Pearson’s chi-squared test or Fisher’s exact test for categorical variables, and unpaired t-test or Mann-Whitney U test for continuous variables. Univariate logistic regression analysis was performed to identify potential predictive factors for MPR/pCR. Clinically relevant factors (age, gender, smoking status, clinical stage, PD-L1 expression) were subsequently entered into multivariate logistic regression analysis to determine independent predictors. Odds ratios (ORs) with 95% CIs were calculated. All statistical analyses were performed using SPSS version 26.0 (IBM Corp., Armonk, NY, USA) and GraphPad Prism version 9.0 (GraphPad Software, San Diego, CA, USA). A two-sided P value < 0.05 was considered statistically significant.

## Results

### Patient characteristics

A total of 137 patients with locally advanced lung squamous cell carcinoma (SCC) who received neoadjuvant chemoimmunotherapy followed by surgical resection were included. The mean age was 64.5 ± 6.1 years. Most patients were male (86.1%, 118/137) and current or former smokers (72.3%, 99/137). ECOG performance status was 1 in 70.1% (96/137) and 0 in 29.9% (41/137). The mean Ki-67 proliferation index was 53.7 ± 20.8%, and the mean PD-L1 tumor proportion score (TPS) was 18.4 ± 22.8%. Regarding clinical stage, 55.5% (76/137) were stage IIIA and 44.5% (61/137) stage IIIB. After neoadjuvant therapy and surgery, 77 patients (56.2%) achieved major pathological response (MPR) or pathological complete response (pCR), while 60 patients (43.8%) were classified as non-MPR (**Table 1**).

**Table 1:** Tumor characteristics of patients with advanced lung SCC.

Characteristic	N%
Age	
Mean ± SEM, years	64.53 ± 6.103
Sex	
Male/female	118/19
ECOG performance status	
0/1	41/96
Smoking status	
Never smoker/ current or former smoker	38/99
PD-L1	18.445 ± 22.808
Ki-67	
Mean ± SEM, (%)	53.678 ± 20.795
T stage	
T1	1 (0.7)
T2	49 (35.8)
T3	55 (40.1)
T4	32 (23.4)
N stage	
N1	19 (13.9)
N2	118 (86.1)
Pathological Tumor Response	
Non-MPR	60 (43.8)
MPR/pCR	77 (56.2)
Clinical Stage	
IIIA	76 (55.5)
IIIB	61 (44.5)

SCC, squamous cell carcinoma; ECOG, eastern cooperative oncology group; T, tumor; N, node; M, metastasis; SEM, standard error of the mean.

### Association between Ki-67 expression and clinicopathological characteristics

**Figure 2:**
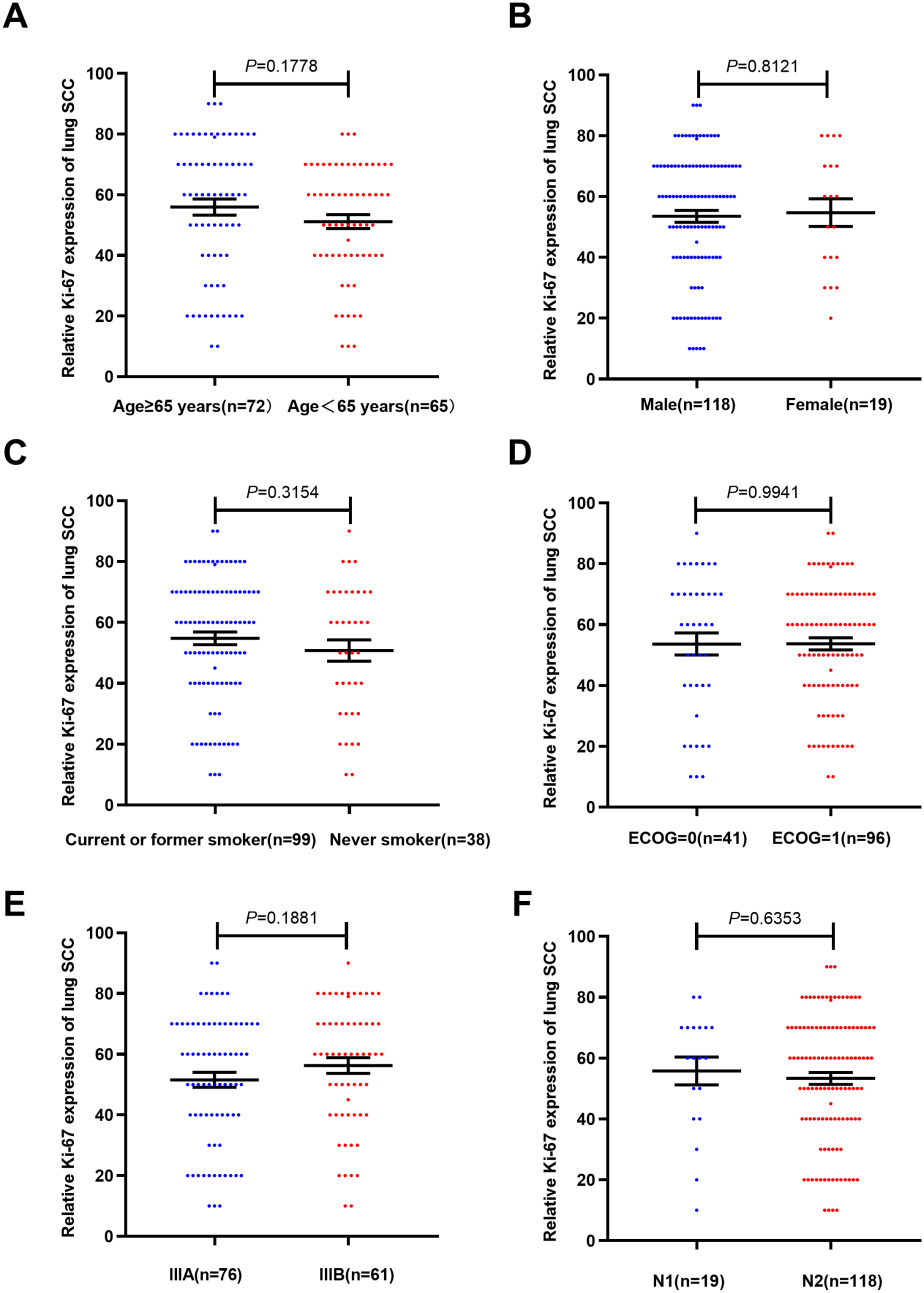
Relationship between Ki-67 expression and clinicopathological characteristics in lung SCC patients receiving neoadjuvant chemoimmunotherapy. **(A)** Age (≥65 years vs. <65 years). **(B)** Gender (Male vs. Female). **(C)** Smoking status (Current or former smoker vs. Never smoker). **(D)** ECOG performance status (0 vs. 1). **(E)** Clinical stage (IIIA vs. IIIB). **(F)** N stage (N1 vs. N2). Horizontal lines represent mean values with standard error of the mean (SEM). P values were calculated using unpaired t-test or Mann-Whitney U test.

No significant associations were observed between Ki-67 expression and age, gender, smoking status, ECOG performance status, clinical stage, or N stage (all P > 0.05), indicating that Ki-67 proliferative index is independent of traditional clinicopathological factors in this cohort (**Figure 2**).

### Ki-67 expression predicts pathological response

**Figure 3:**
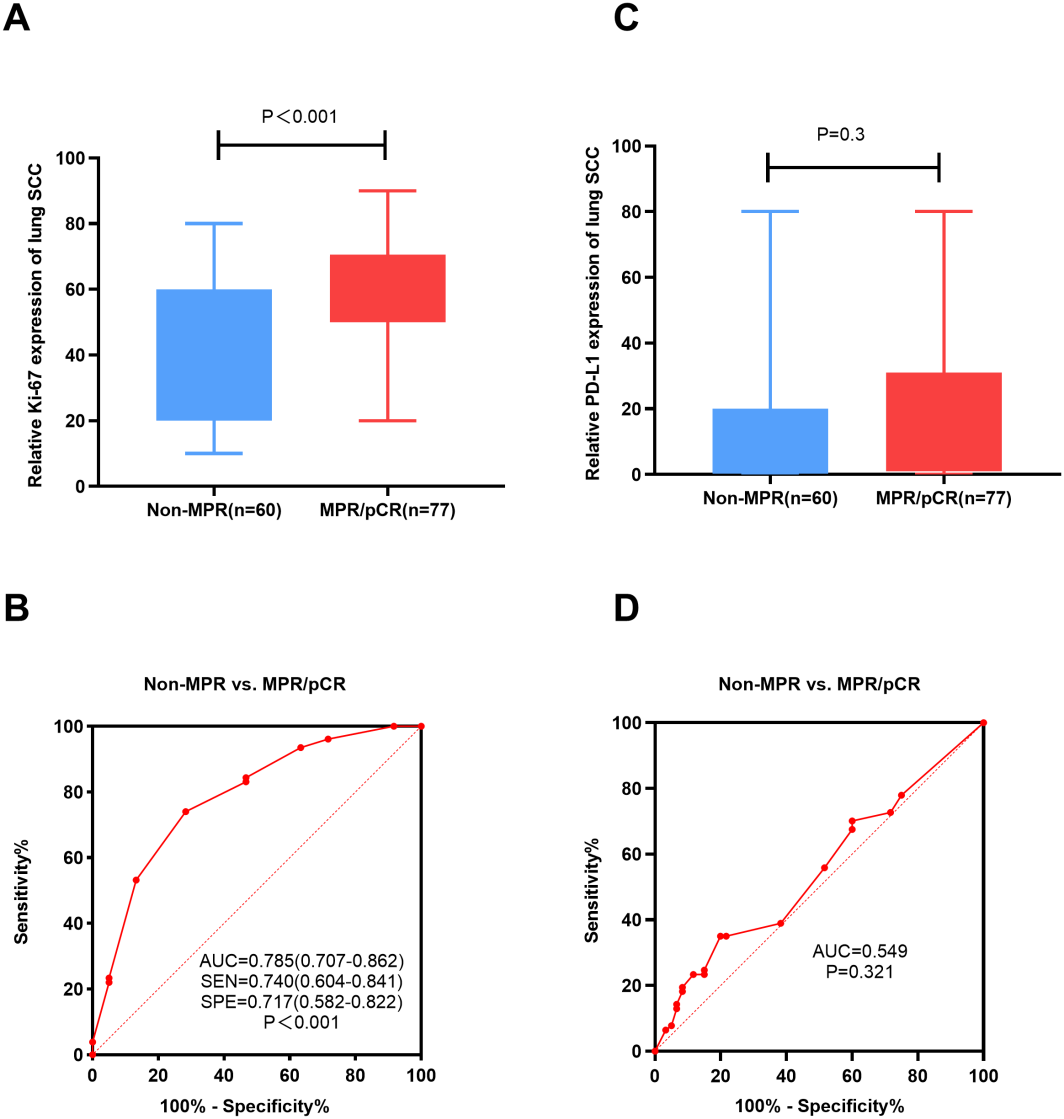
Predictive value of Ki-67 and PD-L1 expression for pathological response in lung SCC patients receiving neoadjuvant chemoimmunotherapy. **(A)** Comparison of Ki-67 expression levels between Non-MPR and MPR/pCR groups (P < 0.001). **(B)** Receiver operating characteristic (ROC) curve analysis showing the predictive accuracy of Ki-67 for MPR/pCR (AUC = 0.785, 95% CI: 0.707–0.862, P < 0.0001). The optimal cutoff value was 55% with a sensitivity of 74.0% and specificity of 71.7%. **(C)** Comparison of PD-L1 expression levels between Non-MPR and MPR/pCR groups (P = 0.3). **(D)** ROC curve analysis showing the predictive accuracy of PD-L1 for MPR/pCR (AUC = 0.549, P = 0.321). Abbreviations: MPR, major pathological response; pCR, complete pathological response; AUC, area under the curve; SEN, sensitivity; SPE, specificity; TPS, tumor proportion score.

Patients who achieved MPR/pCR had significantly higher Ki-67 expression levels compared to those with non-MPR (mean: ~60% vs. ~45%, P < 0.001; **Figure 3A**).ROC curve analysis demonstrated good predictive performance of Ki-67 for MPR/pCR, with an area under the curve (AUC) of 0.785 (95% CI: 0.707–0.862, P < 0.001; **Figure 3B**). The optimal cutoff value determined by the Youden index was 55%, yielding a sensitivity of 74.0% and specificity of 71.7%.

We also constructed a combined predictive model incorporating Ki-67 expression and smoking status. ROC curve analysis showed that this combined model achieved an AUC of 0.804 (95% CI: 0.729–0.879, P < 0.001), representing a modest improvement over Ki-67 alone (AUC = 0.785). These results are presented in **Supplementary Figure 1**.

Using this cutoff, patients with high Ki-67 expression (≥55%) had a significantly higher MPR/pCR rate compared to those with low Ki-67 expression (<55%): 69.6% versus 27.9% (P < 0.001).

By contrast, PD-L1 expression did not differ significantly between the MPR/pCR and non-MPR groups (mean: ~30% vs. ~20%, P = 0.3; **Figure 3C**). Consistently, ROC curve analysis demonstrated poor predictive performance of PD-L1 for MPR/pCR (AUC = 0.549, P = 0.321; **Figure 3D**), further confirming its limited predictive value in the neoadjuvant chemoimmunotherapy setting.

### Independent predictors of MPR/pCR

Univariate logistic regression showed that high Ki-67 expression (≥55%) was significantly associated with MPR/pCR (OR = 7.209, 95% CI: 3.378–15.386, P < 0.001). Smoking status showed a trend toward association (OR = 1.738, P = 0.164), while age, gender, ECOG PS, clinical stage, and PD-L1 expression were not significant (**Table 2**).

**Table 2:** Univariate and multivariate logistic regression analysis of factors associated with major pathological response (MPR) and complete pathological response (PCR).

Risk factors	Univariate analysis			Multivariate analysis		
	OR	95% CI	*P* value	OR	95% CI	*P* value
Gender (Femalevs Male)	0.846	0.320-2.234	0.735	0.241	0.058-1.012	0.052
Age (per year increase)	1.022	0.967-1.080	0.446	0.984	0.916-1.059	0.673
ECOG PS (0 vs 1)	1.529	0.721-3.242	0.268	1.314	0.512-3.371	0.570
Smoking statue (Ever vs Never)	1.738	0.799–3.783	0.164	5.405	1.515-19.280	0.009*
Clinical stage (IIIB vs IIIA)	1.035	0.525-2.040	0.921	1.297	0.567-2.968	0.537
PD-L1 (per 1% increase)	1.011	0.995-1.027	0.175	1.006	0.988-1.025	0.607
Ki-67 (per 1%)	1.060	1.037-1.084	<0.001	1.069	1.043-1.096	<0.001*

*P<0.05. ECOG PS, eastern cooperative oncology group performance status; OR,Odds Ratio; CI, confidence interval.Multivariate model adjusted for all variables listed in the table. N stage and T stage were not significantly associated with MPR/PCR and were excluded from the final model.

In multivariate logistic regression adjusting for all clinicopathological factors, Ki-67 expression (as a continuous variable) remained an independent predictor of MPR/pCR (adjusted OR per 1% increase = 1.069, 95% CI: 1.043–1.096, P < 0.001). Notably, smoking status also emerged as a significant independent predictor (adjusted OR = 5.405, 95% CI: 1.515–19.280, P = 0.009). Gender showed a borderline association (P = 0.052), whereas PD-L1 expression (P = 0.607), clinical stage (P = 0.537), and other factors were not significantly associated with MPR/pCR (**Table 2**).

### Preliminary event-free survival analysis

**Figure 4:**
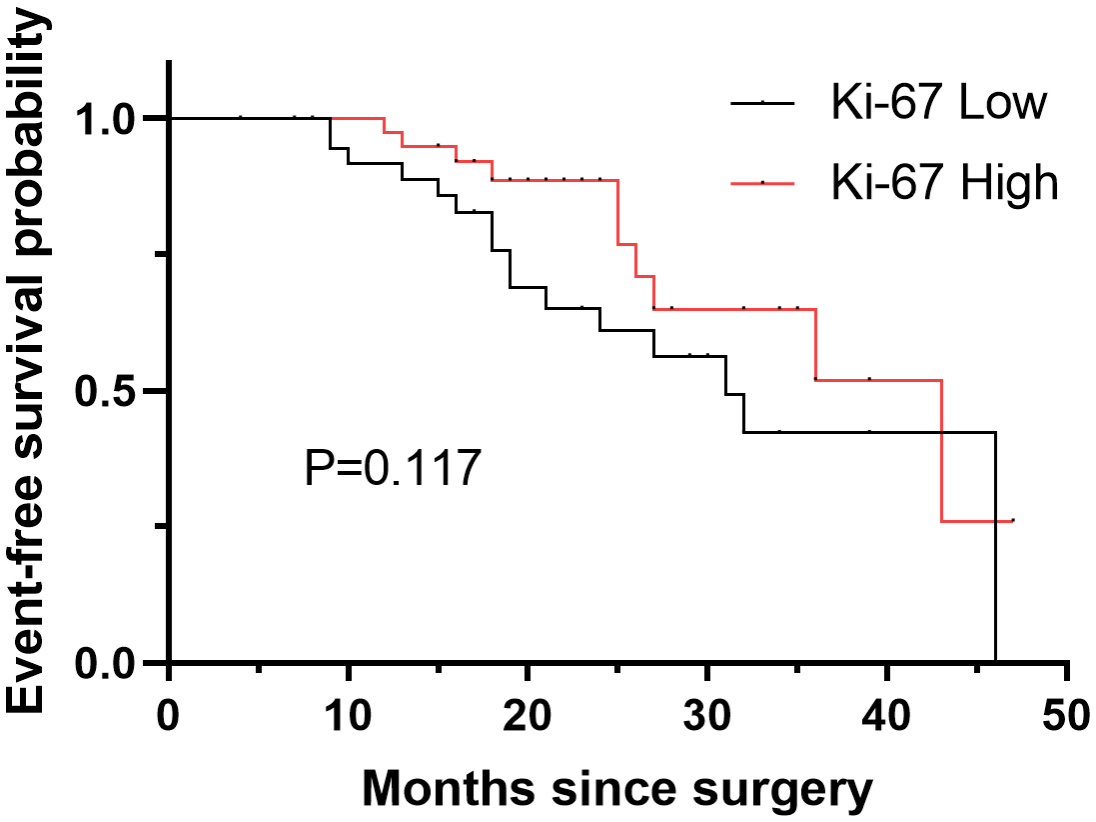
Preliminary event-free survival (EFS) analysis by Ki-67 expression. Kaplan-Meier curves showing EFS in patients with high Ki-67 (≥55%, red line, n=19) versus low Ki-67 (<55%, blue line, n=23). The median EFS was 43.0 months in the high Ki-67 group and 31.0 months in the low Ki-67 group. The difference did not reach statistical significance (log-rank P = 0.117; HR = 0.540, 95% CI: 0.250–1.168).

A subset of 42 patients with complete follow-up data was analyzed for event-free survival (EFS). Based on the Ki-67 cutoff of 55%, patients with high Ki-67 (≥55%, n=19) had a median EFS of 43.0 months, compared to 31.0 months in those with low Ki-67 (<55%, n=23). The difference did not reach statistical significance (log-rank P = 0.117; hazard ratio = 0.540, 95% CI: 0.250–1.168) (**Figure 4**). Tick marks indicate censored observations.

## Discussion

While PD-L1 expression has been established as a predictive biomarker for immunotherapy efficacy in advanced NSCLC, its value in the neoadjuvant setting remains controversial. The LCMC3 trial (9) demonstrated significantly higher MPR rates in patients with PD-L1 TPS ≥50% compared to those with TPS <1% (33% vs. 5%), with baseline PD-L1 levels inversely correlating with the percentage of residual viable tumor cells (R=-0.37, P<0.001), indicating that higher PD-L1 expression predicted deeper pathologic regression. Conversely, both the NEOSTAR trial (10)and the study by Reuss et al. (11)observed that although elevated pretreatment tumor PD-L1 was associated with pathologic response (NEOSTAR: P = 0.037; Reuss et al.: Spearman rho=-0.88, P = 0.02), responses were also noted in patients lacking PD-L1 expression, highlighting the limitations of PD-L1 as a standalone biomarker. In the present study, PD-L1 expression was not significantly associated with pathologic response (P = 0.607), consistent with findings from NEOSTAR, Reuss et al., and the phase II trial by Shu et al. evaluating neoadjuvant atezolizumab plus chemotherapy (MPR 57% irrespective of PD-L1 status, p = 0.67) (12), thereby supporting the need for more refined composite predictive models in the era of neoadjuvant immunotherapy.

While Ki-67 has been extensively validated in breast cancer neoadjuvant therapy, its application varies significantly by treatment modality. Mukai et al. (13)demonstrated that dynamic Ki-67-guided chemotherapy switching in HER2-positive disease actually impaired pCR rates (44.1% vs 23.6%), suggesting limitations of early monitoring in cytotoxic therapy. Conversely, the CARABELA (14)and PETREMAC (15) trials established that baseline or early Ki-67 suppression effectively stratifies patients for endocrine-based de-escalation strategies in HR+/HER2- disease.

In contrast, our study reveals that pretreatment Ki-67 levels (rather than dynamic changes) predict major pathological response (MPR) to neoadjuvant immunotherapy in lung squamous cell carcinoma (AUC = 0.785 at 55% cut-off). This discrepancy likely reflects mechanistic differences: whereas chemotherapy efficacy depends on early cytotoxicity amenable to dynamic monitoring, immunotherapy response is determined by baseline tumor proliferation interacting with immune microenvironment. The higher optimal cut-off (55%) compared to breast cancer standards (30%) underscores the necessity of modality-specific biomarker validation.

In our cohort, smoking status emerged as a significant independent predictor of pathological response (adjusted OR = 5.405, 95% CI: 1.515–19.280, P = 0.009), indicating that smokers exhibited approximately 5.4-fold higher odds of achieving MPR/pCR compared to never-smokers after adjusting for clinicopathological confounders. This finding corroborates the recent meta-analysis by Zhang et al. (16), which demonstrated that smokers derived significant EFS benefit (HR = 0.54, *P* < 0.001) from neoadjuvant PD-1/PD-L1 inhibitors, whereas never-smokers showed no statistically significant benefit (HR = 0.68, *P* = 0.055). The concordance between our multivariate result and their pooled RCT data strengthens the evidence that smoking history serves as a robust predictive biomarker for immunotherapy efficacy in the neoadjuvant setting. Furthermore, our observation aligns with the real-world evidence in advanced NSCLC reported by Yang et al. (17), who identified former heavy smokers (≥20 pack-years) as optimal immunotherapy beneficiaries, exhibiting superior ORR (OR = 1.93, 95% CI: 1.25–2.99) and prolonged PFS (HR = 0.75, 95% CI: 0.57–0.99). The consistency across treatment stages—neoadjuvant in our study versus advanced disease in theirs—suggests that the smoking-associated immune microenvironment, characterized by elevated tumor mutation burden (TMB) and PD-L1 upregulation via the aryl hydrocarbon receptor pathway, universally enhances checkpoint inhibitor efficacy irrespective of treatment timing. It should be emphasized, however, that smoking is not per se a favorable biological characteristic. Instead, smoking history likely serves as an imperfect surrogate marker of underlying tumor immunogenicity, which is driven by elevated tumor mutational burden (TMB) and neoantigen load. Clinicians should avoid interpreting smoking status as a recommendation for or against immunotherapy; rather, it is one of several factors that collectively inform the likelihood of response.

The underlying biological mechanism linking high Ki−67 expression to favorable pathological response may be explained by two interconnected pathways. First, highly proliferative tumor cells with elevated Ki−67 are more vulnerable to cytotoxic chemotherapy, as chemotherapeutic agents predominantly target actively dividing cells. Second, high proliferation activity is associated with increased genomic instability, elevated neoantigen load, and enhanced tumor immunogenicity, which further promote T−cell infiltration and strengthen the efficacy of immune checkpoint inhibitors (7, 18). In line with our results, recent studies have validated that Ki−67 can effectively stratify patients with NSCLC who benefit more from chemoimmunotherapy than from immunotherapy alone (7). Therefore, Ki−67 acts as a dual biomarker reflecting both chemosensitivity and immune activation, which may explain its superior predictive performance over PD−L1 in the neoadjuvant chemoimmunotherapy setting.

Beyond its prognostic and predictive value, Ki-67 serves as a dynamic pharmacodynamic biomarker reflecting tumor proliferative activity that can be modulated by therapeutic interventions. Boyacioglu et al. (19) demonstrated that a novel cannabinoid 1 receptor agonist (ACPA) delivered via polycaprolactone-based nanoparticles significantly suppressed Ki-67 expression in an ectopic NSCLC xenograft model, concomitant with reduced tumor volume and enhanced apoptosis through inhibition of the PI3K/Akt and Ras/MEK/Erk pathways. This finding underscores that high Ki-67 expression, which predicts favorable pathological response to neoadjuvant immunotherapy in our cohort, represents a therapeutically targetable phenotype. Future investigations may explore whether combining neoadjuvant immunotherapy with agents that directly suppress proliferation—such as CB1 agonists or PI3K/Akt inhibitors—could overcome the resistance conferred by elevated Ki-67, potentially improving MPR/pCR rates in patients with high-Ki-67 NSCLC.

Although Ki-67 is an independent predictor, combining it with immune microenvironment markers may enhance accuracy. Omero et al. (20) demonstrated that absolute eosinophil count (AEC) and IL-33-related pathways predict immunotherapy efficacy, suggesting that AI-integrated models incorporating Ki-67, AEC, and cytokine profiles could optimize patient stratification.

In an exploratory EFS analysis, high Ki-67 (≥55%) was associated with a numerically longer median EFS (43.0 vs. 31.0 months), but the difference was not statistically significant (P = 0.117). The wide confidence interval of the HR (0.250–1.168) crossing unity reflects limited statistical power due to the small subset (n=42) and still-immature survival events. It is noteworthy that the direction of effect (HR <1) suggests a potential survival benefit for high Ki-67 patients, which aligns with their significantly higher MPR/pCR rate. This contrasts with some studies in advanced NSCLC where high Ki-67 predicted worse prognosis, highlighting that the predictive and Predictive roles of Ki-67 may differ between the curative neoadjuvant setting and the palliative metastatic setting. Longer follow-up and larger prospective cohorts are needed to definitively establish whether Ki-67 expression can serve as a prognostic biomarker for long-term survival after neoadjuvant chemoimmunotherapy.

This study has several limitations that should be acknowledged. First, this was a retrospective, single−center study with inherent selection bias and potential confounding factors. The conclusions require further validation in prospective, multi−center cohorts with larger sample sizes to improve generalizability. Second, the assessment of Ki−67 expression was based on a single antibody clone and platform, and there is still no universally standardized protocol for Ki−67 testing, scoring, and cutoff determination in lung squamous cell carcinoma. Inter−laboratory and inter−observer variability may affect the reproducibility of results. Third, given the limited sample size of paired pre- and post-treatment specimens, dynamic changes in Ki-67 proliferation index were analyzed as an exploratory endpoint in only three patients and are presented in **Supplementary Figure 2**. Whether early Ki-67 suppression can reliably predict pathological response-–similar to the monitoring strategy in breast cancer-–remains unclear and warrants further investigation in larger cohorts. Fourth, the optimal cutoff value of 55% was derived from the Youden index in this cohort, and prospective validation is needed before routine clinical application. Fifth, this study focused on pathological response (MPR/pCR) as the primary endpoint, and long−term survival outcomes such as disease−free survival (DFS) and overall survival (OS) are still immature; extended follow−up is required to confirm the Predictive value of Ki−67. The preliminary EFS analysis (**Figure 4**) was underpowered due to small sample size and immature follow-up, and the observed numerical advantage in the high Ki-67 group did not reach statistical significance. Extended follow-up is required to determine whether Ki-67 expression predicts long-term survival beyond pathological response. Sixth, due to the retrospective single-center design, we did not employ propensity score matching (PSM) or inverse probability of treatment weighting (IPTW). These methods could potentially balance baseline covariates, but they also reduce sample size (PSM) or rely on model assumptions that may not hold in our relatively modest cohort (n=137). Importantly, our multivariate analysis already adjusted for all major clinicopathological confounders (age, gender, smoking status, clinical stage, PD-L1), and the association between Ki-67 and MPR/pCR remained highly significant (adjusted OR per 1% increase = 1.069, P < 0.001), suggesting that the observed predictive value is robust against measured confounding. Finally, we did not explore the underlying immune microenvironment characteristics or combine Ki−67 with other biomarkers (e.g., TMB, inflammatory indicators) to construct a composite predictive model, which may further improve predictive accuracy.

## Conclusions

In conclusion, pretreatment Ki−67 expression is a robust and independent predictive biomarker for pathological response to neoadjuvant chemoimmunotherapy in locally advanced lung squamous cell carcinoma. Using a cutoff of 55% derived from this cohort, patients with high Ki−67 expression (≥55%) achieved a significantly higher MPR/pCR rate compared to those with low expression. Furthermore, smoking status was identified as an independent predictor of MPR/pCR, with smokers having approximately 5−fold higher odds of response. In contrast, PD−L1 expression showed no significant predictive value in this setting.

These findings suggest that preoperative assessment of Ki−67, together with smoking history, can help identify patients who are more likely to benefit from neoadjuvant chemoimmunotherapy, thereby facilitating individualized treatment strategies. However, given the retrospective, single−center nature of this study and the data−derived cutoff value, prospective multi-center validation is essential before Ki-67 can be incorporated into routine clinical practice for perioperative management of lung squamous cell carcinoma. Although high Ki-67 expression showed a trend toward longer event-free survival, this did not reach statistical significance in the current preliminary analysis; therefore, the predictive value of Ki-67 for long-term survival remains to be confirmed in larger prospective cohorts with mature follow-up.

## Data Availability

The raw data supporting the conclusions of this article will be made available by the authors, without undue reservation.

## Supplementary material

Supplementary Figure 1 Receiver operating characteristic (ROC) curve of the combined predictive model incorporating Ki-67 expression and smoking status for major pathological response (MPR) or pathological complete response (pCR) in patients with locally advanced lung squamous cell carcinoma receiving neoadjuvant chemoimmunotherapy. The area under the curve (AUC) was 0.804 (95% CI: 0.729–0.879, P < 0.001).

Supplementary Figure 2 Dynamic changes of Ki-67 proliferation index in paired pre-treatment biopsy and postoperative surgical specimens from three patients with lung squamous cell carcinoma undergoing neoadjuvant chemoimmunotherapy. **(A, C, E)** Pretreatment Ki-67 immunohistochemical staining in baseline biopsy samples. **(B, D, F)** Corresponding postoperative Ki-67 staining in matched surgical specimens from the same patients. Case 1 **(A, B)**: Ki-67 index changed from __70_% to __30_%; Case 2 **(C, D)**: from _90__% to __50_%; Case 3 **(E, F)**: from _60__% to __5_%. Original magnification, ×400.
