# Supplementary figures and images for: Predictive value of Ki-67 expression in predicting pathological response to neoadjuvant chemotherapy combined with immunotherapy in lung squamous cell carcinoma
Source: Front Oncol. 2026 Jul 7;16:1859319. doi: 10.3389/fonc.2026.1859319 (PMC13384871; doi:10.3389/fonc.2026.1859319)

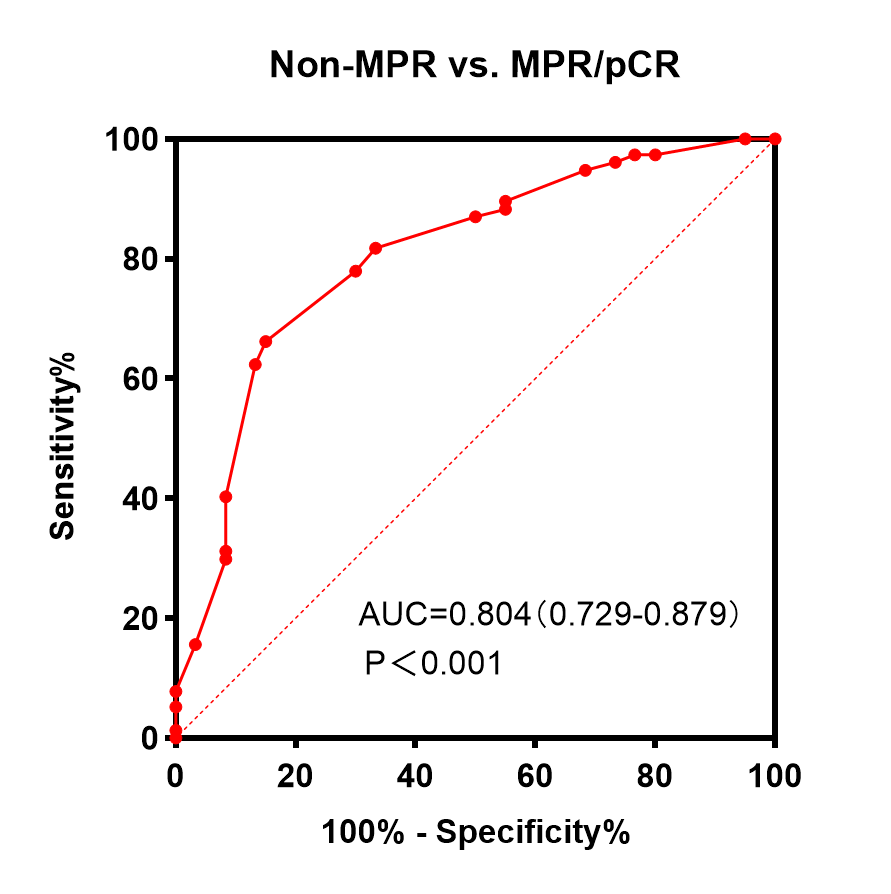

Supplement: Supplementary Figure 1 — Receiver operating characteristic (ROC) curve of the combined predictive model incorporating Ki-67 expression and smoking status for major pathological response (MPR) or pathological complete response (pCR) in patients with locally advanced lung squamous cell carcinoma receiving neoadjuvant chemoimmunotherapy. The area under the curve (AUC) was 0.804 (95% CI: 0.729–0.879, P < 0.001). [file Image1.tif]

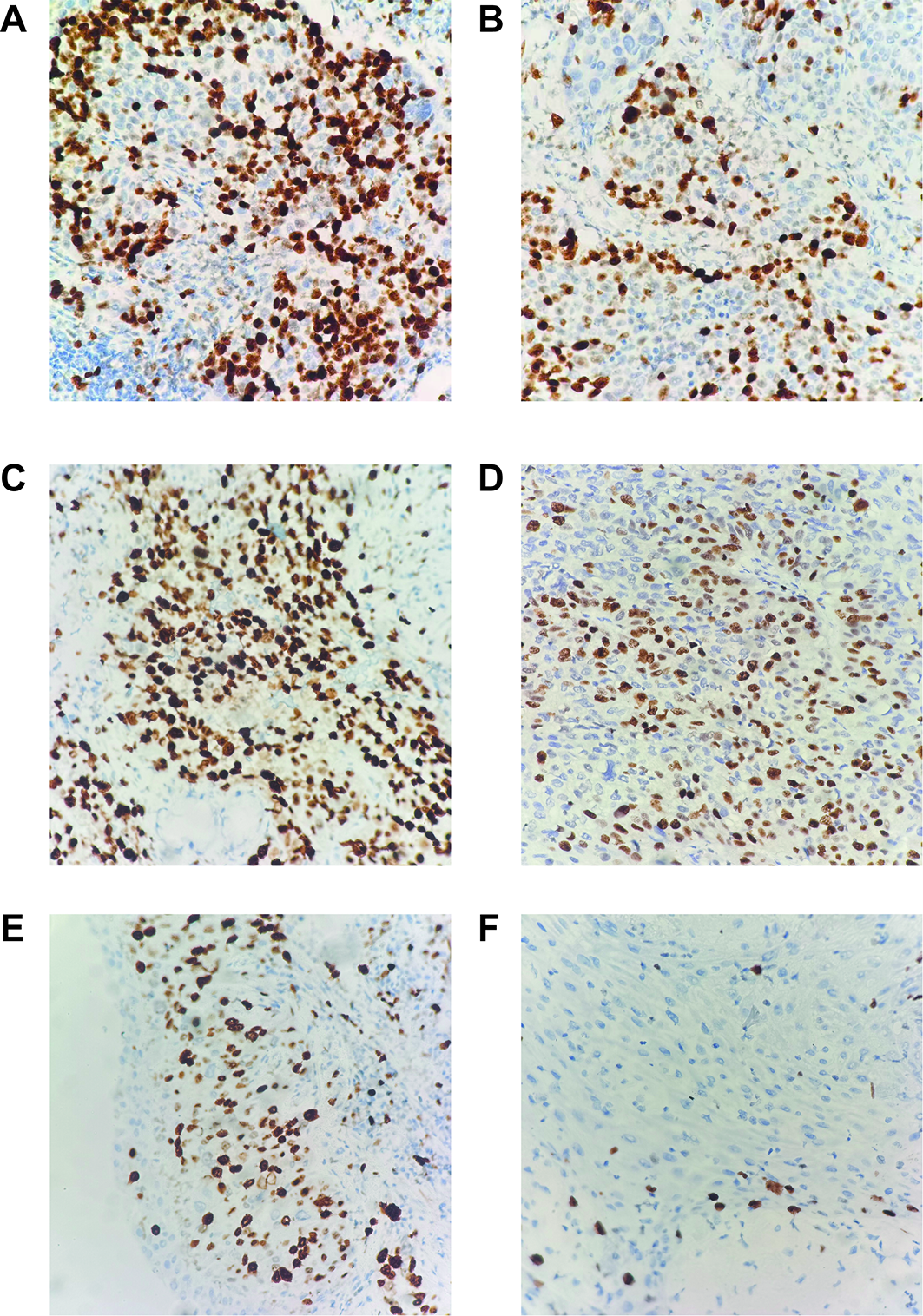

Supplement: Supplementary Figure 2 — Dynamic changes of Ki-67 proliferation index in paired pre-treatment biopsy and postoperative surgical specimens from three patients with lung squamous cell carcinoma undergoing neoadjuvant chemoimmunotherapy. (A, C, E) Pretreatment Ki-67 immunohistochemical staining in baseline biopsy samples. (B, D, F) Corresponding postoperative Ki-67 staining in matched surgical specimens from the same patients. Case 1 (A, B): Ki-67 index changed from __70_% to __30_%; Case 2 (C, D): from _90__% to __50_%; Case 3 (E, F): from _60__% to __5_%. Original magnification, ×400. [file Image2.tif]
